# Crystal structures of dimetal terephthalate di­hydroxides, *M*_2_(C_8_H_4_O_4_)(OH)_2_ (*M* = Co, Ni, Zn) from powder diffraction data and DFT calculations

**DOI:** 10.1107/S2056989022005023

**Published:** 2022-05-13

**Authors:** Emma L. Markun, Drew A. Jensen, Joshua D. Vegetabile, James A. Kaduk

**Affiliations:** a Department of Chemistry, North Central College, 131 S. Loomis, St., Naperville IL, 60540 , USA; University of Aberdeen, Scotland

**Keywords:** powder diffraction, density functional theory, terephthalate, hydroxide, cobalt, nickel, zinc

## Abstract

The crystal structure of poly[di­hydroxido(μ_6_-terepthalato)dizinc], [Zn_2_(C_8_H_4_O_4_)(OH)_2_]_*n*_, was solved and refined using synchrotron powder data, and the structures of the isostructural Co and Ni analogues were refined using laboratory powder X-ray data. The structure of [Co_2_(C_8_H_4_O_4_)(OH)_2_]_*n*_ has been reported previously in space group *C*2/*m*, which yields disordered terephthalate anions. Doubling the *c*-axis of that cell results in an ordered model in space group *C*2/*c*. The octa­hedral *M*O_6_ coordination polyhedra of the metal cations share edges, forming chains running parallel to the *b*-axis direction. These chains share corners (hydroxyl groups), forming layers lying perpendicular to the *a*-axis direction.

## Abstract 2

A new ordered *C*2/*c* model is proposed for the dimetal terephthalate di­hydroxides *M* = Co, Ni, and Zn, compared to the previous disordered *C*2/*m* model for *M* = Co.

## 1. Chemical context

Dicobalt terephthalate di­hydroxide, Co_2_(C_8_H_4_O_4_)(OH)_2_, was first prepared by Sherif (1970 ▸). A powder pattern was reported, but no unit cell or crystal structure were determined. The powder pattern from this reference is included in the Powder Diffraction File (Gates-Rector & Blanton, 2019 ▸) as entry 00-034-1897. A search of the nine peaks of this entry against the PDF-4 Organics 2022 database yielded no additional terephthalate compounds. 
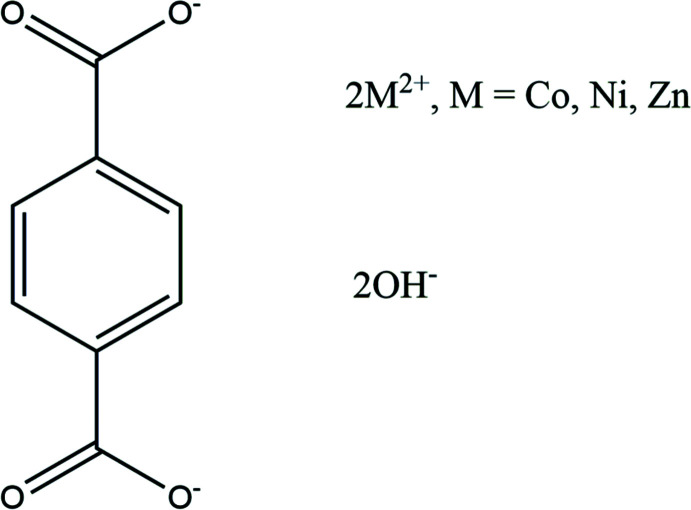


Approximately 20 years ago, one of us (JAK) solved and refined the structure of Zn_2_(C_8_H_4_O_4_)(OH)_2_ using synchrotron powder data, first in a *C*2/*m* cell with disordered terephthalate anions. It then became apparent that if the *c*-axis were doubled, the systematic absences corresponded to space group *C*2/*c*. This doubled unit cell removed the disorder and yielded a more satisfactory refinement. This structure was deposited in the Cambridge Structural Database (Kaduk, 2016 ▸; refcode PUCYAO01), but never otherwise published or discussed. Since that time, another polymorph of Zn_2_(C_8_H_4_O_4_)(OH)_2_ (in space group *P*2_1_/*c*) has been reported (Carton *et al.*, 2009 ▸; PUCYAO).

Some of our recent attempts to prepare Co and Ni porous metal–organic frameworks (MOFs) yielded instead cobalt and nickel terephthalate hydroxide. We took advantage of the opportunity to re-refine the structures (as well as that of Zn) in what we believe to be the correct space group, and to optimize the structures using density functional techniques.

## 2. Structural commentary

**Figure 1:**
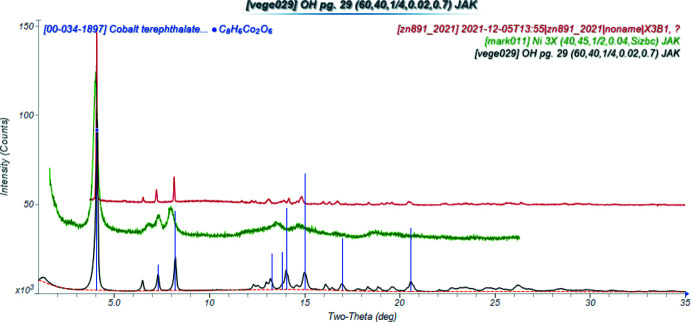
The X-ray powder diffraction patterns of Co_2_(C_8_H_4_O_4_)(OH)_2_ (black), Ni_2_(C_8_H_4_O_4_)(OH)_2_ (green), and Zn_2_(C_8_H_4_O_4_)(OH)_2_ (red). The Zn pattern (measured using Co radiation) and the Zn pattern (measured using synchrotron radiation) were converted to the Mo wavelength used to measure the Co pattern using *JADE Pro* (MDI, 2021 ▸).

**Figure 2:**
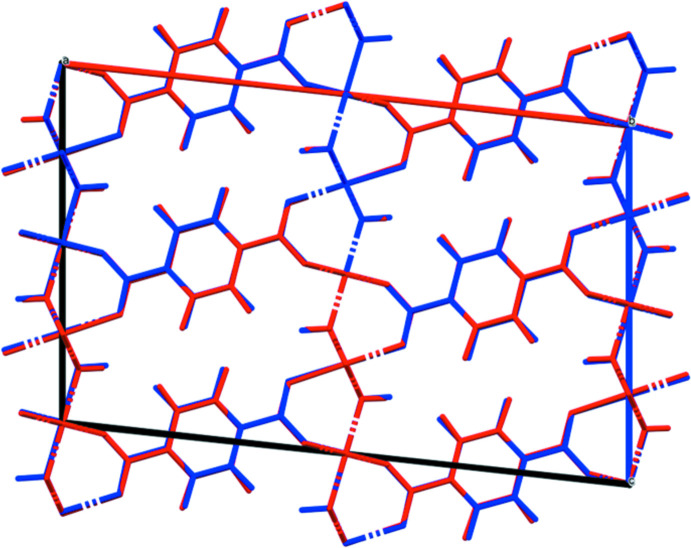
Comparison of the Rietveld-refined (red) and VASP-optimized (blue) structures of Co_2_(C_8_H_4_O_4_)(OH)_2_. The r.m.s. Cartesian displacement is 0.125 Å.

**Figure 5:**
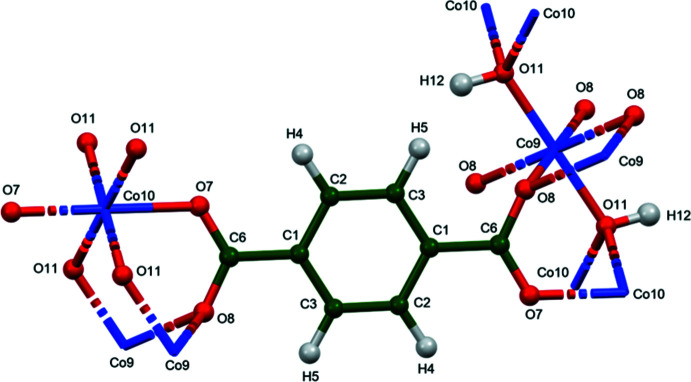
The asymmetric unit of Co_2_(C_8_H_4_O_4_)(OH)_2_, with the atom numbering. The atoms are represented by 50% probability spheroids.

**Figure 6:**
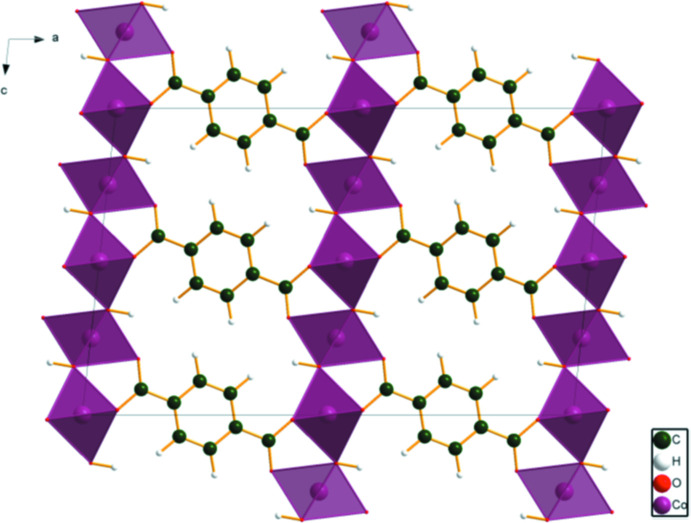
The crystal structure of Co_2_(C_8_H_4_O_4_)(OH)_2_, viewed down the *b*-axis direction.

**Figure 7:**
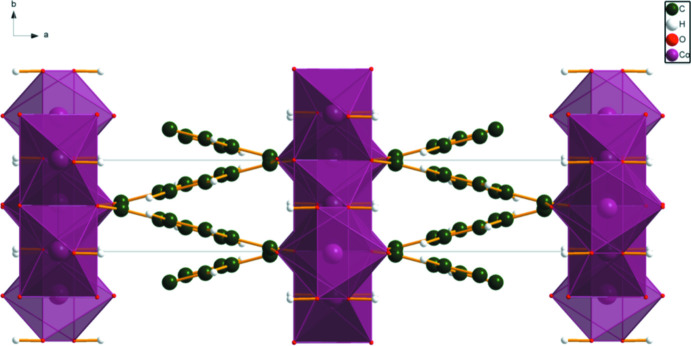
The crystal structure of Co_2_(C_8_H_4_O_4_)(OH)_2_, viewed down the *c*-axis direction.

Doubling the *c*-axis of the previously reported disordered *C*2/*m* model for Co results in a chemically-reasonable ordered *C*2/*c* structure for these compounds. The X-ray powder diffraction patterns show that the three compounds are isostructural (Fig. 1 ▸). The root-mean-square Cartesian displacements of the non-H atoms in the Rietveld-refined and DFT-optimized structures are 0.125, 0.143, and 0.339 Å for Co, Ni, and Zn, respectively (Figs. 2 ▸–4 ▸ ▸). The good agreement provides strong evidence that the structures are correct (van de Streek & Neumann, 2014 ▸). This discussion concentrates on the DFT-optimized structures. The asymmetric unit (with atom numbering) is illustrated in Fig. 5 ▸. The best view of the crystal structure is down the *b*-axis (Fig. 6 ▸). A view down the *c*-axis is shown in Fig. 7 ▸.

Almost all of the bond distances, angles, and torsion angles in the terephthalate anions fall within the normal ranges indicated by a *Mercury* Mogul Geometry check (Macrae *et al.*, 2020 ▸). Only the Ni9—O11 bond distance of 2.187 Å [average = 2.007 (9) Å, *Z*-score = 20.4] and the Zn14—O16 bond of 1.970 Å [average = 2.122 (47) Å, *Z*-score = 3.2] are flagged as unusual. The carbox­yl–phenyl torsion angles of 7.5, 9.8, and 6.2° for Co, Ni, and Zn, respectively, correspond to a distortion energy of only ∼2 kJ mol^−1^ (Kaduk *et al.*, 1999 ▸). This energy penalty can easily be compensated for by coordination to the cations. The closest Miller plane of the phenyl ring is (85 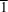 ) for Co and Ni, and (530) for Zn. *M*9 lies on a center of symmetry, and *M*10 lies on a twofold axis. For M = Co, Co9 has two shorter Co—O distances of 2.000 Å, and four longer ones ∼2.19–2.20 Å. Co10 has four distances ∼2.11 Å, and two at 2.157 Å. For *M* = Ni, all six distances to Ni9 are 2.187–2.232 Å, and Ni10 has four shorter distances at 2.03–2.08 Å and two longer at 2.115 Å. For *M* = Zn, Zn9 has two short distances of 1.969 Å, and four long ones at ∼2.22 Å whereas Zn10 has two distances of 2.095 Å and four at 2.14–2.18 Å. Both Co9 and Co10 exhibit octa­hedral coordination. The coordination sphere of Co9 contains two *trans* O7 and four equatorial O11 (hydroxyl group), while Co10 has two *trans* O11 and four equatorial O8. The hydroxyl group bridges three cobalt atoms: one Co9 and two Co10. Atom O7 coordinates to Co10, and O8 bridges two Co9 atoms; as a result each carboxyl group bridges three metal atoms. The bond-valence sums (Brown, 2002 ▸) are 1.90 and 1.84 for Co9 and Co10, respectively, 1.78 and 1.93 for Ni9 and Ni10, and 1.92 and 1.86 for Zn9 and Zn10. All cations are thus slightly under-bonded compared to their expected values of 2.00.

The peak profiles are dominated by microstrain broadening. The generalized microstrain model was used for Co and Zn, but the limited Ni data supported refinement of only an isotropic broadening coefficient. The average microstrain is similar for Co and Zn (21042 and 20094 ppm, respectively), while that for Ni is much larger, at 114830 ppm. Perhaps this greater microstrain indicates that some square-planar Ni coordination also occurs. Analysis of the contributions to the total crystal energy of the structure using the Forcite module of *Materials Studio* (Dassault Systèmes, 2021 ▸) suggests that for Co and Ni, the bond and angle distortion terms dominate intra­molecular deformation energy, but that torsion terms are also significant. For Zn, the angle distortion terms dominate the intra­molecular deformation energy. The inter­molecular energy in all three compounds is dominated by electrostatic attractions, which represent the *M*—O bonds.

The Bravais–Friedel–Donnay–Harker (Bravais, 1866 ▸; Friedel, 1907 ▸; Donnay & Harker, 1937 ▸) morphology suggests that we might expect elongated (with [010] as the long axis) or platy (with {200} as the major faces) morphology for these compounds. A 2nd order spherical harmonic model was included in the refinement. The texture indices were 1.003, 1.417, and 1.016 for Co, Ni, and Zn respectively, showing that preferred orientation was significant only for the flat-plate Ni specimen.

## 3. Supra­molecular features

**Figure 8:**
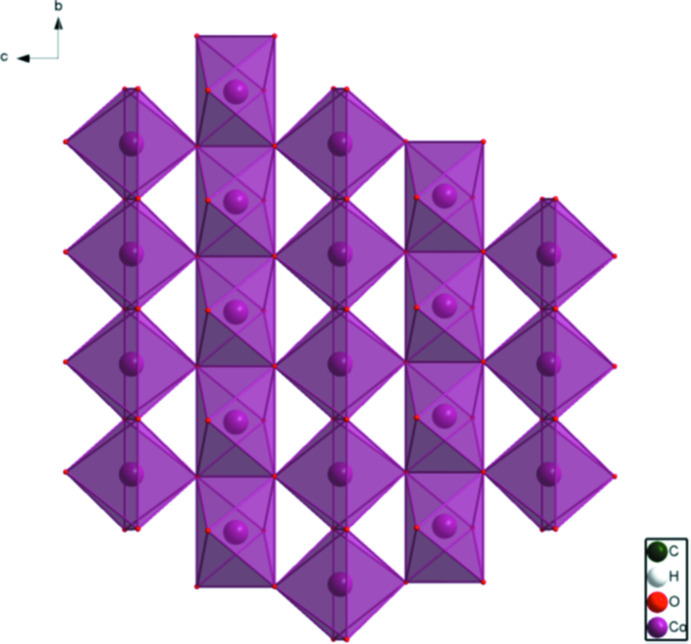
The layers in the crystal structure of Co_2_(C_8_H_4_O_4_)(OH)_2_, viewed down the *a*-axis direction.

The octa­hedral coordination spheres of Co9 share edges, forming chains running parallel to the *b*-axis direction; the shared edges are parallel the *a*-axis direction. The octa­hedral coordination spheres of Co10 share edges, forming chains propagating along the *b*-axis; in this case, the shared edges lie parallel to the *c*-axis direction. Co9 and Co10 share corners (*via* O11 = the hydroxyl group), forming layers lying perpendicular to the *a*-axis direction (Fig. 8 ▸). The hydroxyl group does not participate in hydrogen bonds.

## 4. Database survey

The crystal structure of the ‘new terephthalate-based cobalt hydroxide Co_2_(OH)_2_(C_8_H_4_O_4_)’ was reported by Huang *et al.* (2000 ▸), and its crystal structure determined [Cambridge Structural Database (Groom *et al.*, 2016 ▸) refcode QASLIF] by *ab initio* methods using X-ray powder diffraction data. The reported space group is *C*2/*m* with *a* = 19.943 (1), *b* = 3.2895 (1), *c* = 6.2896 (3) Å, *β* = 95.746 (3)°, *V* = 410.545 Å^3^, and *Z* = 2. The structure consists of alternating Co-hydroxide and terephthalate layers, and the terephthalate anions are disordered about an inversion center. Anti­ferromagnetic ordering in this compound was studied using neutron powder diffraction by Feyerherm *et al.* (2003 ▸), using the same unit cell (QASLIF02). The structure was also determined by Kurmoo *et al.* (2001 ▸; QASLIF01) in the same unit cell, as well as the structure of cobalt terephthalate dihydrate. The structures of a series of (Co,Fe)_2_(C_8_H_4_O_4_)(OH)_2_ solid solutions were refined in the same unit cell by Mesbah *et al.* (2010 ▸) (UJIMOQ, UJIMOQ01, UJINAD, UJINAD01) using synchrotron and neutron powder data. A reduced cell search in the Cambridge Structural Database yielded in addition the structures of Ni_2_(C_8_H_4_O_4_)(OH)_2_ (Mesbah *et al.*, 2014 ▸, NIWQOB; Han *et al.*, 2018 ▸, NIWQOB01).

## 5. Synthesis and crystallization

Cobalt(II) nitrate hexa­hydrate (0.0364 g, 0.125 mmol) and terephthalic acid (0.0208 g, 0.125 mmol) were added to a flask followed by 0.125 ml of tri­ethyl­amine and approximately 5 ml of di­methyl­formamide. The reaction was stirred for 10 min until a homogenous mixture was obtained. The reaction was heated using a CEM Discover microwave with power set to 150 W using a ramp time of 2 min to reach 423 K with a hold time of 30 min and inter­nal stirring switched off. The vial remained in the microwave until it cooled to 323 K, and the reaction mixture was filtered using vacuum filtration, washed with DMF and deionized water (10 ml each). The remaining solid was dried in an oven at 343 K under vacuum.

Nickel(II) nitrate hexa­hydrate (0.1948 g, 0.67 mmol) and terephthalic acid (0.2492 g, 1.5 mmol) were dissolved in 10 ml of DMF in a microwave vial. The solution was stirred until homogenous. The solution was then heated using a CEM Mars 6 microwave reactor at 750 W for a total of 85 s, in increments of 25 and 60 s. The resulting green solid was isolated using vacuum filtration, washed with water, methanol, and acetone, and allowed to air dry.

Information on the synthesis of Zn_2_(C_8_H_4_O_4_)(OH)_2_ from prior to 1997 is no longer available.

## 6. Refinement

Crystal data, data collection and structure refinement details are summarized in Table 1 ▸.

**Figure 9:**
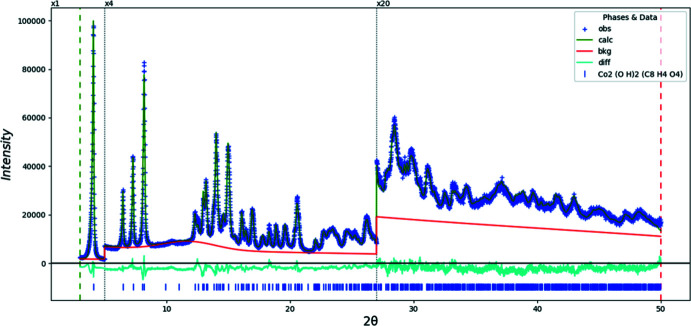
The Rietveld plot for the refinement of Co_2_(C_8_H_4_O_4_)(OH)_2_. The blue crosses represent the observed data points, and the green line is the calculated pattern. The cyan curve is the normalized error plot. The row of tick marks indicates the calculated reflection positions. The vertical scale has been multiplied by a factor of 4× for 2θ > 5.0°, and by a factor of 20× for 2θ > 27.0°.

Rietveld refinements (Figs. 9 ▸–11 ▸ ▸) were carried out using *GSAS-II* (Toby & Von Dreele, 2013 ▸). All non-H bond distances and angles in the terephthalate anions were subjected to restraints, based on a *Mercury* Mogul Geometry Check (Sykes *et al.*, 2011 ▸; Bruno *et al.*, 2004 ▸). The Mogul average and standard deviation for each qu­antity were used as the restraint parameters. The restraints contributed 0–2.3% to the final χ^2^. The *U*_iso_ were grouped by chemical similarity. The *U*_iso_ values for the H atoms were fixed at 1.3 × the *U*_iso_ of the heavy atoms to which they are attached. The peak profiles were described using the generalized microstrain model. The background was modeled using a 3–12-term shifted Chebyshev polynomial.

The structures were optimized with density functional techniques using VASP (Kresse & Furthmüller, 1996 ▸) (fixed experimental unit cells) through the MedeA graphical inter­face (Materials Design, 2016 ▸). The calculations were carried out on 16 2.4 GHz processors (each with 4 Gb RAM) of a 64-processor HP Proliant DL580 Generation 7 Linux cluster at North Central College. The calculations for Co and Ni were spin-polarized magnetic calculations, using the simplified LDSA + U approach, and *U*_j_ = 3.7 eV for Co and Ni. The calculations used the GGA-PBE functional, a plane wave cutoff energy of 400.0 eV, and a *k*-point spacing of 0.5 Å^−1^ leading to an 8 × 8 × 2 mesh.

## Supplementary Material

Crystal structure: contains datablock(s) Co_Riet, Co_DFT, Ni_Riet, Ni_DFT, Zn_Riet, Zn_DFT. DOI: 10.1107/S2056989022005023/hb8022sup1.cif

CCDC references: 2171929, 2171928, 2171927, 2171926, 2171925, 2171924

Additional supporting information:  crystallographic information; 3D view; checkCIF report

## Figures and Tables

**Figure 3:**
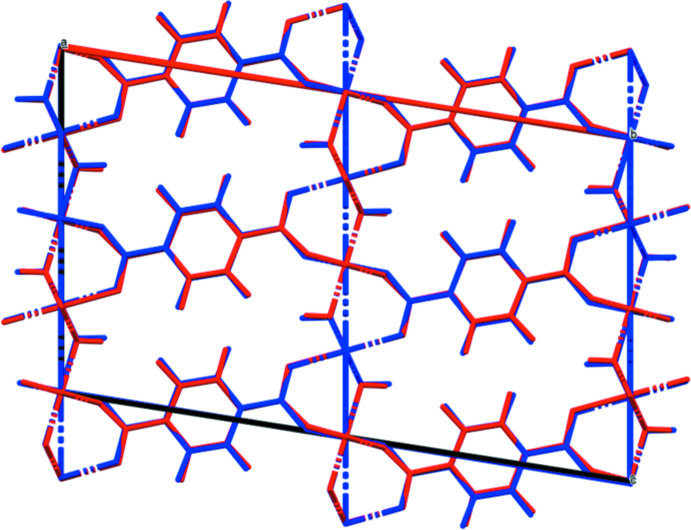
Comparison of the Rietveld-refined (red) and VASP-optimized (blue) structures of Ni_2_(C_8_H_4_O_4_)(OH)_2_. The r.m.s. Cartesian displacement is 0.143 Å.

**Figure 4:**
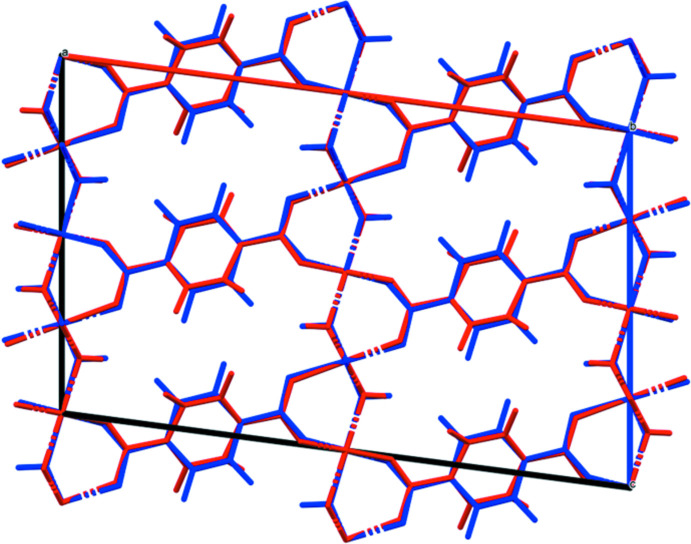
Comparison of the Rietveld-refined (red) and VASP-optimized (blue) structures of Zn_2_(C_8_H_4_O_4_)(OH)_2_. The r.m.s. Cartesian displacement is 0.339 Å.

**Figure 10:**
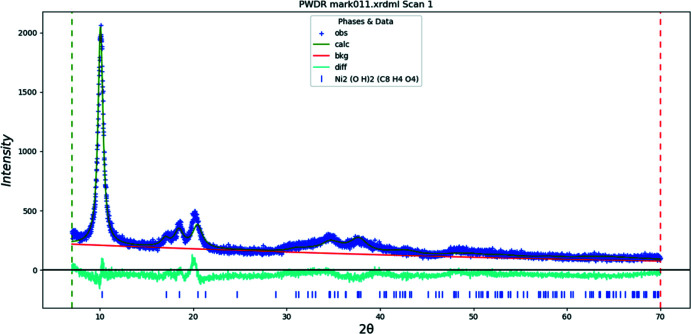
The Rietveld plot for the refinement of Ni_2_(C_8_H_4_O_4_)(OH)_2_. The blue crosses represent the observed data points, and the green line is the calculated pattern. The cyan curve is the normalized error plot. The row of tick marks indicates the calculated reflection positions.

**Figure 11:**
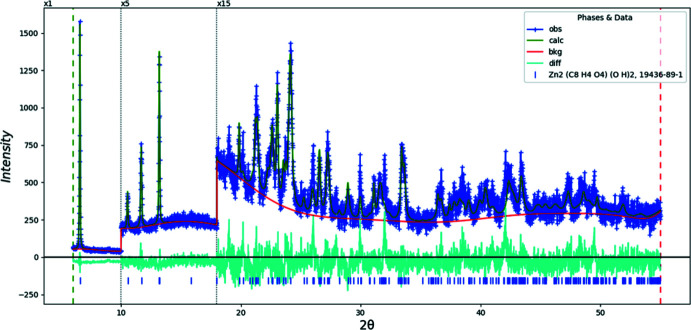
The Rietveld plot for the refinement of Zn_2_(C_8_H_4_O_4_)(OH)_2_. The blue crosses represent the observed data points, and the green line is the calculated pattern. The cyan curve is the normalized error plot. The row of tick marks indicates the calculated reflection positions. The vertical scale has been multiplied by a factor of 5× for 2θ > 10.0°, and by a factor of 15× for 2θ > 18.0°.

**Table 1:** Experimental details

	[Co_2_(C_8_H_4_O_4_)(OH)_2_]	[Ni_2_(C_8_H_4_O_4_)(OH)_2_]	[Zn_2_(C_8_H_4_O_4_)(OH)_2_]
Crystal data			
*M*_r_	316	315.53	328.89
Crystal system, space group	Monoclinic, *C*2/*c*	Monoclinic, *C*2/*c*	Monoclinic, *C*2/*c*
Temperature (K)	300	300	300
*a*, *b*, *c* (Å)	19.9554 (10), 3.2883 (2), 12.6139 (8)	20.35 (5), 3.364 (6), 12.19 (4)	20.165 (2), 3.3273 (5), 12.5956 (16)
β (°)	96.059 (5)	98.9 (2)	97.431 (10)
*V* (Å^3^)	823.08 (6)	824.6 (15)	837.99 (14)
*Z*	4	4	4
Radiation type	Mo *K*α_1,2_, λ = 0.70932, 0.71361 Å	Co *K*α_1,2_, λ = 1.78892, 1.79278 Å	Synchrotron, λ = 1.15008 Å
Specimen shape, size (mm)	Cylinder, 12 × 0.7	Flat sheet, 16 × 16	Cylinder, ? × ?

Data collection			
Diffractometer	PANalytical Empyrean	PANalytical X’Pert	NSLS beamline X3B1
Specimen mounting	Glass capillary	Si zero-background plate with well	Kapton capillary
Data collection mode	Transmission	Reflection	Transmission
Scan method	Step	Step	Step
2θ values (°)	2θ_min_ = 1.002, 2θ_max_ = 49.991, 2θ_step_ = 0.008	2θ_min_ = 4.007, 2θ_max_ = 69.983, 2θ_step_ = 0.017	2θ_min_ = 6.0, 2θ_max_ = 60.0, 2θ_step_ = 0.01

Refinement			
*R* factors and goodness of fit	*R*_p_ = 0.045, *R*_wp_ = 0.063, *R*_exp_ = 0.020, *R*(*F* ^2^) = 0.05751, χ^2^ = 10.414	*R*_p_ = 0.084, *R*_wp_ = 0.107, *R*_exp_ = 0.070, *R*(*F* ^2^) = 0.14454, χ^2^ = 2.369	*R*_p_ = 0.092, *R*_wp_ = 0.121, *R*_exp_ = 0.097, *R*(*F* ^2^) = 0.14121, χ^2^ = 1.573
No. of parameters	42	12	57
No. of restraints	15	0	14
(Δ/σ)_max_	0.025	97.398	1.459

The same symmetry and lattice parameters were used for the DFT calculations as for each powder diffraction study. Computer programs: *GSAS-II* (Toby & Von Dreele, 2013 ▸), *Mercury* (Macrae *et al.*, 2020 ▸), *DIAMOND* (Crystal Impact, 2015 ▸), and *publCIF* (Westrip, 2010 ▸).

## supplementary crystallographic information

### Poly[dihydroxido(µ6-terepthalato)dicobalt] (Co_Riet) Crystal data

**Table:**

[Co_2_(C_8_H_4_O_4_)(OH)_2_]	*V* = 823.08 (6) Å^3^
*M**_r_* = 316	*Z* = 4
Monoclinic, *C*2/*c*	*D*_x_ = 2.550 Mg m^−^^3^
*a* = 19.9554 (10) Å	Mo *K*α_1,2_ radiation, λ = 0.70932, 0.71361 Å
*b* = 3.2883 (2) Å	*T* = 300 K
*c* = 12.6139 (8) Å	pink
β = 96.059 (5)°	cylinder, 12 × 0.7 mm

### Poly[dihydroxido(µ6-terepthalato)dicobalt] (Co_Riet) Data collection

**Table:**

PANalytical Empyrean diffractometer	Scan method: step
Specimen mounting: glass capillary	2θ_min_ = 1.002°, 2θ_max_ = 49.991°, 2θ_step_ = 0.008°
Data collection mode: transmission	

### Poly[dihydroxido(µ6-terepthalato)dicobalt] (Co_Riet) Refinement

**Table:**

Least-squares matrix: full	42 parameters
*R*_p_ = 0.045	15 restraints
*R*_wp_ = 0.063	H-atom parameters not defined?
*R*_exp_ = 0.020	Weighting scheme based on measured s.u.'s
*R*(*F*^2^) = 0.05751	(Δ/σ)_max_ = 0.025
5864 data points	Background function: Background function: "chebyschev-1" function with 4 terms: 1063(5), -577(6), 95(4), -25(3), Background peak parameters: pos, int, sig, gam: 11.866, 3892.401, 44425.907, 0.100,
Profile function: Finger-Cox-Jephcoat function parameters U, V, W, X, Y, SH/L: peak variance(Gauss) = Utan(Th)^2^+Vtan(Th)+W: peak HW(Lorentz) = X/cos(Th)+Ytan(Th); SH/L = S/L+H/L U, V, W in (centideg)^2^, X & Y in centideg 30.816, 10.768, 0.000, 1.935, 0.000, 0.033, Crystallite size in microns with "isotropic" model: parameters: Size, G/L mix 1.000, 1.000, Microstrain, "generalized" model (10^6^ * delta Q/Q) parameters: S400, S040, S004, S220, S202, S022, S301, S103, S121, G/L mix 2180.060, 4.385767395e6, 5373.300, 103711.383, 724.789, 689333.161, -2196.502, 2609.389, 91248.973, 0.800,	Preferred orientation correction: Simple spherical harmonic correction Order = 2 Coefficients: 0:0:C(2,-2) = -0.0542; 0:0:C(2,0) = -0.1055; 0:0:C(2,2) = -0.0207

### Poly[dihydroxido(µ6-terepthalato)dicobalt] (Co_Riet) Fractional atomic coordinates and isotropic or equivalent isotropic displacement parameters (Å^2^)

**Table:**

	*x*	*y*	*z*	*U*_iso_*/*U*_eq_	
C1	0.3140 (2)	0.106 (3)	0.0412 (4)	0.018 (3)*	
C2	0.2695 (3)	0.269 (3)	0.1093 (3)	0.0184*	
C3	0.2024 (3)	0.368 (4)	0.0686 (4)	0.0184*	
H4	0.28873	0.24618	0.19840	0.0220*	
H5	0.17120	0.48125	0.13117	0.0220*	
C6	0.3837 (3)	−0.024 (5)	0.0845 (5)	0.0200*	
O7	0.3988 (3)	−0.007 (7)	0.1799 (5)	0.020000*	
O8	0.4268 (3)	−0.031 (5)	0.0168 (5)	0.020000*	
Co9	0.50000	0.50000	0.50000	0.0020 (5)*	
Co10	0.00000	0.495 (3)	−0.25000	0.0020*	
O11	0.0287 (3)	0.030 (7)	0.1569 (7)	0.0200*	
H12	0.06922	−0.03114	0.16621	0.0260*	

### Poly[dihydroxido(µ6-terepthalato)dicobalt] (Co_Riet) Geometric parameters (Å, º)

**Table:**

C1—C2	1.406 (6)	O8—C6	1.274 (4)
C1—C3^i^	1.391 (3)	O8—Co9^ii^	2.151 (12)
C1—C6	1.501 (5)	Co9—O8^iii^	2.151 (12)
C2—C1	1.406 (6)	Co9—O8^iv^	2.151 (12)
C2—C3	1.421 (4)	Co9—O11^v^	2.004 (8)
C3—C1^i^	1.391 (3)	Co9—O11^vi^	2.004 (8)
C3—C2	1.421 (4)	Co10—O7^i^	2.119 (5)
C6—C1	1.501 (5)	Co10—O7^vii^	2.119 (5)
C6—O7	1.211 (5)	Co10—O11^viii^	2.072 (16)
C6—O8	1.274 (4)	Co10—O11^ix^	2.072 (16)
O7—C6	1.211 (5)	O11—Co9^x^	2.004 (8)
O7—Co10^i^	2.119 (5)	O11—Co10^viii^	2.072 (16)
			
C2—C1—C3^i^	119.1 (3)	C1^i^—C3—C2	119.2 (3)
C2—C1—C6	120.4 (2)	C1—C6—O7	118.2 (4)
C3^i^—C1—C6	119.5 (3)	C1—C6—O8	115.2 (5)
C1—C2—C3	119.9 (3)	O7—C6—O8	123.5 (6)

Symmetry codes: (i) −*x*+1/2, −*y*+1/2, −*z*; (ii) −*x*+1, *y*−1, −*z*+1/2; (iii) −*x*+1, *y*+1, −*z*+1/2; (iv) *x*, −*y*, *z*+1/2; (v) −*x*+1/2, *y*+1/2, −*z*+1/2; (vi) *x*+1/2, −*y*+1/2, *z*+1/2; (vii) *x*−1/2, −*y*+1/2, *z*−1/2; (viii) −*x*, −*y*+1, −*z*; (ix) *x*, −*y*+1, *z*−1/2; (x) −*x*+1/2, *y*−1/2, −*z*+1/2.

### (Co_DFT) Crystal data

**Table:**

C_8_H_6_Co_2_O_6_	*c* = 12.59800 Å
*M**_r_* = 316	β = 96.33°
Monoclinic, *C*2/*c*	*V* = 828.49 Å^3^
*a* = 20.02520 Å	*Z* = 4
*b* = 3.30420 Å	

### (Co_DFT) Data collection

**Table:**

*h* = →	*l* = →
*k* = →	

### (Co_DFT) Fractional atomic coordinates and isotropic or equivalent isotropic displacement parameters (Å^2^)

**Table:**

	*x*	*y*	*z*	*U*_iso_*/*U*_eq_	
C1	0.31569	0.14758	0.04012	0.0184	
C2	0.26806	0.23812	0.11006	0.0184	
C3	0.20287	0.33955	0.07035	0.0184	
H4	0.28329	0.23013	0.19578	0.022	
H5	0.16565	0.41266	0.12436	0.022	
C6	0.38562	0.03773	0.08352	0.020	
O7	0.39861	0.98679	0.18301	0.020	
O8	0.42960	0.00198	0.01583	0.020	
Co9	0.00000	0.00000	0.00000	0.002	
Co10	0.50000	0.97237	0.25000	0.002	
O11	0.02838	0.97997	0.15714	0.020	
H12	0.07726	0.97414	0.17088	0.026	

### Poly[dihydroxido(µ6-terepthalato)dinickel] (Ni_Riet) Crystal data

**Table:**

[Ni_2_(C_8_H_4_O_4_)(OH)_2_]	*V* = 824.6 (15) Å^3^
*M**_r_* = 315.53	*Z* = 4
Monoclinic, *C*2/*c*	*D*_x_ = 2.542 Mg m^−^^3^
*a* = 20.35 (5) Å	Co *K*α_1,2_ radiation, λ = 1.78892, 1.79278 Å
*b* = 3.364 (6) Å	*T* = 300 K
*c* = 12.19 (4) Å	pale green
β = 98.9 (2)°	flat_sheet, 16 × 16 mm

### Poly[dihydroxido(µ6-terepthalato)dinickel] (Ni_Riet) Data collection

**Table:**

PANalytical X'Pert diffractometer	Scan method: step
Specimen mounting: Si zero-background plate with well	2θ_min_ = 4.007°, 2θ_max_ = 69.983°, 2θ_step_ = 0.017°
Data collection mode: reflection	

### Poly[dihydroxido(µ6-terepthalato)dinickel] (Ni_Riet) Refinement

**Table:**

Least-squares matrix: full	12 parameters
*R*_p_ = 0.084	0 restraints
*R*_wp_ = 0.107	H-atom parameters not defined?
*R*_exp_ = 0.070	(Δ/σ)_max_ = 97.398
*R*(*F*^2^) = 0.14454	Background function: Background function: "chebyschev-1" function with 3 terms: 139.3(6), -71.0(8), 7.7(7),
3949 data points	Preferred orientation correction: Simple spherical harmonic correction Order = 2 Coefficients: 0:0:C(2,-2) = -0.91(8); 0:0:C(2,0) = 0.63(8); 0:0:C(2,2) = 0.93(13)
Profile function: Finger-Cox-Jephcoat function parameters U, V, W, X, Y, SH/L: peak variance(Gauss) = Utan(Th)^2^+Vtan(Th)+W: peak HW(Lorentz) = X/cos(Th)+Ytan(Th); SH/L = S/L+H/L U, V, W in (centideg)^2^, X & Y in centideg 2.761, 0.000, 1.090, 3.610, 0.000, 0.047,	

### Poly[dihydroxido(µ6-terepthalato)dinickel] (Ni_Riet) Fractional atomic coordinates and isotropic or equivalent isotropic displacement parameters (Å^2^)

**Table:**

	*x*	*y*	*z*	*U*_iso_*/*U*_eq_	
C1	0.31564	0.11999	0.03425	0.0100*	
C2	0.27426	0.26556	0.10759	0.0100*	
C3	0.20930	0.40008	0.07333	0.0100*	
H4	0.28873	0.24618	0.19840	0.0130*	
H5	0.17120	0.48125	0.13117	0.0130*	
C6	0.38661	−0.01497	0.07632	0.0100*	
O7	0.39933	−0.00110	0.17831	0.0100*	
O8	0.43321	−0.00173	0.01302	0.0100*	
Ni9	0.50000	0.50000	0.50000	0.0387*	
Ni10	0.00000	0.50145	−0.25000	0.0387*	
O11	0.02771	0.0007	0.15823	0.0063*	
H12	0.06922	−0.03114	0.16621	0.0082*	

### Poly[dihydroxido(µ6-terepthalato)dinickel] (Ni_Riet) Geometric parameters (Å, º)

**Table:**

C1—C2	1.4074	O8—C6	1.3123
C1—C3^i^	1.3331	Ni9—O11^ii^	1.9246
C1—C6	1.5251	Ni9—O11^iii^	1.9246
C2—C1	1.4074	Ni10—O7^i^	2.0995
C2—C3	1.3985	Ni10—O7^iv^	2.0995
C3—C1^i^	1.3331	Ni10—O11^v^	2.149
C3—C2	1.3985	Ni10—O11^vi^	2.1377
H5—C3	1.1584	Ni10—O11^vii^	2.149
C6—C1	1.5251	Ni10—O11^viii^	2.1377
C6—O7	1.2313	O11—Ni9^ix^	1.9246
C6—O8	1.3123	O11—Ni10^v^	2.149
O7—C6	1.2313	O11—Ni10^vi^	2.1377
O7—Ni10^i^	2.0995		
			
C2—C1—C3^i^	117.925	C1^i^—C3—C2	118.448
C2—C1—C6	121.216	C1—C6—O7	111.649
C3^i^—C1—C6	120.856	C1—C6—O8	121.645
C1—C2—C3	123.601	O7—C6—O8	122.291

Symmetry codes: (i) −*x*+1/2, −*y*+1/2, −*z*; (ii) −*x*+1/2, *y*+1/2, −*z*+1/2; (iii) *x*+1/2, −*y*+1/2, *z*+1/2; (iv) *x*−1/2, −*y*+1/2, *z*−1/2; (v) −*x*, −*y*, −*z*; (vi) −*x*, −*y*+1, −*z*; (vii) *x*, −*y*, *z*−1/2; (viii) *x*, −*y*+1, *z*−1/2; (ix) −*x*+1/2, *y*−1/2, −*z*+1/2.

### (Ni_DFT) Crystal data

**Table:**

C_8_H6Ni_2_O_6_	*c* = 12.22464 Å
*M**_r_* = 315.53	β = 99.20°
Monoclinic, *C*2/*c*	*V* = 805.74 Å^3^
*a* = 20.40719 Å	*Z* = 4
*b* = 3.27188 Å	

### (Ni_DFT) Data collection

**Table:**

*h* = →	*l* = →
*k* = →	

### (Ni_DFT) Fractional atomic coordinates and isotropic or equivalent isotropic displacement parameters (Å^2^)

**Table:**

	*x*	*y*	*z*	*U*_iso_*/*U*_eq_	
C1	0.31659	0.14671	0.03597	0.010	
C2	0.27424	0.23178	0.11288	0.010	
C3	0.20831	0.33316	0.07742	0.010	
H4	0.29416	0.22005	0.20095	0.013	
H5	0.17519	0.40045	0.13678	0.013	
C6	0.38690	0.03143	0.07591	0.010	
O7	0.90294	0.45891	0.17836	0.010	
O8	0.42774	0.00936	0.00489	0.010	
Ni9	0.00000	0.00000	0.00000	0.03866	
Ni10	0.00000	0.45204	0.25000	0.03866	
O11	0.52764	0.45960	0.16035	0.00631	
H12	0.57607	0.45471	0.17494	0.00821	

### Poly[dihydroxido(µ6-terepthalato)dizinc] (Zn_Riet) Crystal data

**Table:**

[Zn_2_(C_8_H_4_O_4_)(OH)_2_]	*V* = 837.99 (14) Å^3^
*M**_r_* = 328.89	*Z* = 4
Monoclinic, *C*2/*c*	*D*_x_ = 2.607 Mg m^−^^3^
*a* = 20.165 (2) Å	Synchrotron radiation, λ = 1.15008 Å
*b* = 3.3273 (5) Å	*T* = 300 K
*c* = 12.5956 (16) Å	white
β = 97.431 (10)°	

### Poly[dihydroxido(µ6-terepthalato)dizinc] (Zn_Riet) Data collection

**Table:**

NSLS beamline X3B1 diffractometer	Scan method: step
Specimen mounting: Kapton capillary	2θ_min_ = 6.0°, 2θ_max_ = 60.0°, 2θ_step_ = 0.01°
Data collection mode: transmission	

### Poly[dihydroxido(µ6-terepthalato)dizinc] (Zn_Riet) Refinement

**Table:**

Least-squares matrix: full	57 parameters
*R*_p_ = 0.092	14 restraints
*R*_wp_ = 0.121	H-atom parameters not defined?
*R*_exp_ = 0.097	(Δ/σ)_max_ = 1.459
*R*(*F*^2^) = 0.14121	Background function: Background function: "chebyschev-1" function with 12 terms: 28.78(11), -16.88(18), 8.18(16), 0.45(16), -2.60(15), -1.04(14), 3.60(13), -1.88(13), 0.46(12), 1.97(12), -1.94(11), 1.26(10),
5400 data points	Preferred orientation correction: Simple spherical harmonic correction Order = 2 Coefficients: 0:0:C(2,-2) = -0.05(4); 0:0:C(2,0) = -0.18(6); 0:0:C(2,2) = -0.21(4)
Profile function: Finger-Cox-Jephcoat function parameters U, V, W, X, Y, SH/L: peak variance(Gauss) = Utan(Th)^2^+Vtan(Th)+W: peak HW(Lorentz) = X/cos(Th)+Ytan(Th); SH/L = S/L+H/L U, V, W in (centideg)^2^, X & Y in centideg 6.427, -1.067, 0.000, 0.000, 0.000, 0.022, Crystallite size in microns with "isotropic" model: parameters: Size, G/L mix 1.000, 1.000, Microstrain, "generalized" model (10^6^ * delta Q/Q) parameters: S400, S040, S004, S220, S202, S022, S301, S103, S121, G/L mix 807.414, 6.074702219e6, 12850.425, 116093.843, 1080.871, 214564.056, 1450.184, -4276.159, -164837.348, 0.600,	

### Poly[dihydroxido(µ6-terepthalato)dizinc] (Zn_Riet) Fractional atomic coordinates and isotropic or equivalent isotropic displacement parameters (Å^2^)

**Table:**

	*x*	*y*	*z*	*U*_iso_*/*U*_eq_	
C1	0.3153 (9)	0.132 (11)	0.038 (2)	0.148 (18)*	
C2	0.2801 (13)	0.369 (13)	0.1020 (17)	0.148*	
C3	0.2145 (14)	0.477 (16)	0.067 (2)	0.148*	
H7	0.29840	0.55800	0.17260	0.192*	
H8	0.18320	0.73700	0.09150	0.192*	
C11	0.3863 (5)	0.016 (8)	0.0750 (18)	0.0500*	
O12	0.4045 (9)	0.025 (16)	0.1743 (18)	0.050000*	
O13	0.4201 (7)	−0.156 (9)	0.008 (2)	0.050000*	
Zn14	0.50000	0.50000	0.50000	0.078 (4)*	
Zn15	0.00000	0.54200	−0.25000	0.078*	
O16	0.0318 (13)	0.072 (14)	0.161 (3)	0.0500*	
H17	0.06922	−0.03114	0.16621	0.065000*	

### Poly[dihydroxido(µ6-terepthalato)dizinc] (Zn_Riet) Geometric parameters (Å, º)

**Table:**

C1—C2	1.3877 (16)	O13—C11	1.284 (2)
C1—C3^i^	1.43 (4)	O13—Zn14^ii^	1.989 (13)
C1—C11	1.498 (3)	Zn14—O13^iii^	1.989 (13)
C2—C1	1.3877 (16)	Zn14—O13^iv^	1.989 (13)
C2—C3	1.385 (2)	Zn14—O16^v^	2.06 (3)
C3—C1^i^	1.43 (4)	Zn14—O16^vi^	2.06 (3)
C3—C2	1.385 (2)	Zn15—O12^i^	2.048 (17)
C11—C1	1.498 (3)	Zn15—O12^vii^	2.048 (17)
C11—O12	1.2573 (14)	Zn15—O16^viii^	1.87 (4)
C11—O13	1.284 (2)	Zn15—O16^ix^	1.87 (4)
O12—C11	1.2573 (14)	O16—Zn14^x^	2.06 (3)
O12—Zn15^i^	2.048 (17)	O16—Zn15^viii^	1.87 (4)
			
C2—C1—C3^i^	119.81 (12)	C1—C11—O12	116.6 (2)
C2—C1—C11	120.4 (2)	C1—C11—O13	118.59 (19)
C3^i^—C1—C11	119.6 (4)	O12—C11—O13	123.42 (15)
C1—C2—C3	120.20 (17)	O16^viii^—Zn15—O16^xi^	93 (2)
C1^i^—C3—C2	119.83 (12)		

Symmetry codes: (i) −*x*+1/2, −*y*+1/2, −*z*; (ii) −*x*+1, *y*−1, −*z*+1/2; (iii) −*x*+1, *y*+1, −*z*+1/2; (iv) *x*, −*y*, *z*+1/2; (v) −*x*+1/2, *y*+1/2, −*z*+1/2; (vi) *x*+1/2, −*y*+1/2, *z*+1/2; (vii) *x*−1/2, −*y*+1/2, *z*−1/2; (viii) −*x*, −*y*+1, −*z*; (ix) *x*, −*y*+1, *z*−1/2; (x) −*x*+1/2, *y*−1/2, −*z*+1/2; (xi) *x*, −*y*+1, *z*+1/2.

### (Zn_DFT) Crystal data

**Table:**

C_8_H_6_O_6_Zn_2_	*c* = 12.59470 Å
*M**_r_* = 328.89	β = 97.52°
Monoclinic, *C*2/*c*	*V* = 837.00 Å^3^
*a* = 20.15960 Å	*Z* = 4
*b* = 3.32510 Å	

### (Zn_DFT) Data collection

**Table:**

*h* = →	*l* = →
*k* = →	

### (Zn_DFT) Fractional atomic coordinates and isotropic or equivalent isotropic displacement parameters (Å^2^)

**Table:**

	*x*	*y*	*z*	*U*_iso_*/*U*_eq_	
C1	0.31537	0.14052	0.03973	0.14798	
C2	0.26900	0.22410	0.11036	0.14798	
C3	0.20398	0.33320	0.07087	0.14798	
H7	0.28504	0.20470	0.19624	0.19231	
H8	0.16745	0.40076	0.12504	0.19231	
C11	0.38529	0.02740	0.08247	0.050	
O12	0.39938	0.97473	0.18247	0.050	
O13	0.42745	0.99381	0.01434	0.050	
O16	0.02778	0.97322	0.15570	0.050	
H17	0.07652	0.96489	0.16886	0.065	
Zn14	0.00000	0.00000	0.00000	0.07842	
Zn15	0.50000	0.96690	0.25000	0.07842	
